# Modification of implant surfaces to stimulate mesenchymal cell activation

**DOI:** 10.1186/s42269-022-00743-x

**Published:** 2022-03-04

**Authors:** Ilma Robo, Saimir Heta, Dhimitri Papakozma, Vera Ostreni

**Affiliations:** 1 Faculty of Dental Medicine, University of Medicine, Tiranë, Albania; 2 Pediatric Surgery, Pediatric Surgeon, University Hospital, QSUT, Tiranë, Albania; 3 Private Dental Clinic, Tiranë, Albania; 4 Department of Morphology, University of Medicine, Tiranë, Albania

**Keywords:** Mesenchymal cell, Implant surface, Differentiation

## Abstract

**Background:**

The process of osteointegration, as key point has the activation of mesenchymal cells at implant-bone interspace, their differentiation into osteoblasts and connection between the implant surface and the surrounding bone.

**Main text:**

Implant surfaces composed by biocompatible, organism-friendly materials require changes in content and surface morphology; changes that may further stimulate mesenchymal cell activation. The way the implant surfaces are affected with advantages and disadvantages, that typically bring each methodology, is also the purpose of this study. The study is of review type, based on finding articles about implant surface modification, with the aim of promoting the mesenchymal cell activation, utilizing keyword combination.

**Conclusions:**

Implant success beyond the human element of the practicioner and the protocol element of implant treatment, also relies on the application of the right type of implant, at the right implant site, in accordance with oral and individual health status of the patient. Implant success does not depend on type of "coating" material of the implants. Based at this physiological process, the success or implant failure is not a process depending on the type of selected implant, because types of synthetic or natural materials that promote osteointegration are relatively in large number.

## Background

Mesenchymal cells with individual differentiation potential are the essential elements of possible applications of tissue engineering, with focus on regenerative medicine. These cells found in different areas of the body, regardless of location, are multipotent self-renewing cells, with the potential for multipotent differentiation into osteoblasts, adipocytes and chondroblasts. They are normally found in bone marrow, in dental pulp, in umbilical cord and placenta, in adipose tissue, in menstrual fluids, but each type exhibite individual potential for differentiation and phenotype (Alcayaga-Miranda et al. 2017; Prockop et al. 2010; Billing et al. 2016). According to Commission of the International Association for Therapy with Mesenchymale Cells, the minimum criteria for determining human MSCs are: (a) the application not to the plastic surface; (b) specific expression of surface antigen (positive expression of CD105, CD73 and CD90, and absence of CD45, CD34, CD14 or CD11b, CD79a or CD19 and HLA-DR); and (c) the potential for multipotent in vitro differentiation into osteoblasts, adipocytes, and chondroblasts, using standard differentiating tissue culture conditions. Their diversity for damaged tissues, privileged immune status, and lower risk of tumorigenesis, make them an interesting tool at cell-based therapy. Due to the plasticity of differentiation, immunoregulatory properties, angiogenic modulation and paracrine support, MSCs have been investigated in a wide range of disease indicators, which have been identified in 500 trials recorded in the ClinicalTrials.gov NIH database (http://www.clinicaltrials.gov/, December 2016) (Alcayaga-Miranda et al. 2017; Prockop et al. 2010; Billing et al. 2016).

Mesenchymal cells are cells with the ability to differentiate in an organized way into a functional cell network. This is the reason why mesenchymal cells are used in various medical applications. Mesenchymal cells have multipotential proliferative capacity and are capable differentiating into cartilage, bone, neural cells and adipocytes. Signaling pathways, transcription factors, and growth factors modulate the differentiation of mesenchymal cells into different functional cell lines. Even physical factors can influence the choice of differentiation pathway. These signals include: bone morphogenic proteins (BMPs), epidermal growth factors (EGFs), transformative growth factors (TGFs), integral area wingless proteins (wnt), fibroblastic growth factors (FGFs), and transcriptional regulation (Bhaskar et al. 2014).

Mesenchymal cells have direct and indirect effects on process of differentiation into osteoblasts and chondrocytes. A variety of documented and hypothetical factors exist as decisive in this differentiation process. These factors arise from modulation of transcription factors that are specific for differentiation into osteoblasts and chondrocytes. In bone fractures, as well as in cases of implant osteointegration, mesenchymal cells contribute to bone healing, through direct and indirect effects, producing cytokines, growth factors and vascularization regulators and inflammatory modulators.

The direct structural and functional connection between bone and surface of a functional implant with occlusal force overloaded is called osteointegration. Osteointegrated implants are used for treatment of edentulousness and for the reconstruction of head and neck, to facilitate auricular mandibular, maxillary, nasal and orbital tension, according to implants placed with these specifics (Parnia et al. 2018; Zhao et al. 2009).

Titanium (Ti) and its compounds are widely used for orthopedic and dental implants, due to their excellent mechanical properties and superior biocompatibility. Limited corrosion resistance, insufficient osteintegration and peri-implant infections are the reasons for implant failure (Herranz-Diez et al. 2016). Corrosion and scratch resistance are relatively reduced, which leads to degradation of mechanical properties and aseptic loss of intraoral placed implant (Godoy-Gallardo et al. 2014). The relatively slow osteointegration of titanium implants leads to extension of healing time, to a delay during the process of occlusal implant overloading which leads further at implant clinical failure. Tendencies to increase corrosion resistance and increase osteogenic activity (Xie et al. 2005; Cao et al. 2013) are critical, at effective modifications of implant surface production technique, which may amplify these simultaneous effects.

## Main text

The study is of review type, with the aim of finding out how the modification of the implant surface affects the promotion of activation and subsequent differentiation of mesenchymal cells. The period selected for e-research is the interval 2011–2019, based at the simple reason that COVID-19 Era has its own influences on the advantages of science research in orientations and empowerment with research funds.

The study is of review type using the keywords: mesenchymal cells implant surface, differentiation, and their combination with the aim of finding logical connections between the activity of mesenchymal cells and possible and different modifications of the implant surface. The search was conducted in several attempts to find articles, (Esfahanian et al. 2012) where gradually from 259 articles it was narrowed the list at 73 articles and after the elimination of articles in accordance with the criteria of non-inclusion and during the reading and analysis phase of abstracts, at the end of the electronic search, was a total of 36 articles, available for further and detailed analysis, in accordance with the purpose of the study (Omar et al. 2011; Wang et al. 2014; Herranz-Diez et al. 2016; Chang et al. 2016; Hyzy et al. 2017; D'Alimonte et al. 2017; Guillem-Marti et al. 2019; Smaranda Dana Buduru 2019; Bressel et al. 2017; Yusa et al. 2011, 2016; Galli et al. 2013; Burghardt et al. 2015; Lauria et al. 2018; Zhou and Zhao 2016; Yu et al. 2017; Manfredi et al. 2016; Calzado-Martín et al. 2011; Wei et al. 2019; Singhatanadgit et al. 2019; Kwon and Park 2018; Sagomonyants et al. 2011; Maleki-Ghaleh et al. 2015; Yang et al. 2015; Hao et al. 2016; He et al. 2016; Zhou et al. 2017; Ping et al. 2017; Li et al. 2017; Zheng et al. 2017; Tsuchiya et al. 2018; Shao et al. 2018; Chen et al. 2018; Deng et al. 2018; An et al. 2018; Karthik et al. 2013; Adell et al. 1981; Heta and Robo 2018; Bouri et al. 2008; Banche et al. 2007; Klokkevold and Han 2007; Koldsland et al. 2009; Robo et al. 2017; Alsaadi et al. 2007; Olate et al. 2010; Baqain et al. 2012; Jacobi-Gresser et al. 2013; Brånemark et al. 1999; Branemark et al. 2001; Vendramini et al. 2021).

Criteria of non-inclusion in further analysis of collected items oriented the electronic search to exclude items based on data collection from less than 10 clinical cases in patients. When data on effectiveness of methodology and protocols, indicated by different types of implants are from experiments performed both in vitro and in vivo and are based in large samples, it tends to come to obvious quantitative conclusions. This is the logical explanation of the first criterion of non-inclusion.

The second criterion was the exclusion of all case-report items, as a single clinical case may be a “spark” to further initiate research into large samples, but may never be significant for success or failure, of the implant manufactured by a particular firm with the specific modification indicated. However, this methodology of work at electronic search for articles is also based on previous applications in the already published literature (An et al. 2018).

Ethics and Consent to Participate: As the authors of the article, we state that there is no violation of the code of ethics during the realization of this article. The local ethics committee ruled that no formal ethics approval was required in this particular case. This study was submitted to and approved by Albanian University Institutional Ethics Committee, date 02.07.2019, Tirana, Albania, according to national regulations.

After processing the results, the data are presented in the tables below. Table 1 divides the articles according by which way the implant success is analyzed, by in vitro or in vivo experiments, according to the time intervals of years.

**Table 1:** Number of articles expressed in percentages, divided by type of experiment performed and the year of publication

Year of publication	Type of article		
	In vitro (No-%)	In vivo (No-%)	Total (No-%)
2011–2013	4–11%	0–0%	4–11%
2014–2016	9–25%	4–11%	13–36%
2017–2019	13–36%	6–17%	19–53%
Total	26–72%	10–28%	36–100%

The way experiments are performed with the aim to collect different data about the response of mesenchymal cells at various modifications of implant surface, divides published articles depending on the way the experiment was performed, in rats or humans, in laboratory or in vivo. These data are presented in Table 2.

**Table 2:** The table shows in several articles the trend of publications about experiments performed on rats or humans, in vivo or in vitro

Type of article Application	In vitro			In vivo		
	2011–2013	2014–2016	2016–2019	2011–2013	2014–2016	2016–2019
At rats	2	2	2	0	2	4
At humans	2	7	11	0	2	2
Total	4–11%	9–25%	13–36%	0–0%	4–11%	6–17%

Modification of the implant surface occurs in different ways depending on the “brand” of produced implant. These modifications are presented in Table 3.

**Table 3:** The type of experiments performed on rats or humans, in vivo or in vitro, is related to the way how the implant surface was modified, according to the methods presented in the table

Type of article Modification	In vitro (No-%)	In vivo (No-%)	Total (No-%)
Protein recombinant	5–14%	3–8%	8–22%
Laser	2–5%	0–0%	2–5%
Ions: Zn, Co, Sr, Mg, Li	5–14%	2–5%	7–19%
Titanium nanotubes	5–14%	0–0%	5–14%
Different substances	9–25%	4–11%	13–36%
Ultrasound	0–0%	1–3%	1–3%
Total	26–72%	10–28%	36–100%

Below are listed the 36 selected articles based on the study criteria.

For recombinant proteins, studies were performed by: Omar et al. (2011), Wang et al. (2014), Herranz–Diez et al. (2016). Chang et al. (2016), Hyzy et al. (2017), D’Alimonte et al. (2017). Guillem-Marti et al. (2019), Smaranda Dana Buduru et al. (D'Alimonte et al. 2017).

Articles about the positive reaction of the application of laser at the implant surfaces for the activation and proliferation of mesenchymal cells are: in vitro study in 2016, by Sisti KE et al., which evaluated the modification of the laser implant surface and the ability to interact with mesenchymal cells on this modification, and article by Bressel et al. (2017), who published data about in vitro application of laser-modified human implant surface.

The application of different ions, as another successful method for promoting mesenchymal cell proliferation has been supported by articles of in vitro studies: Yusa et al. (2011, 2016), Galli et al. (2013), Burghardt et al. (2015), Lauria et al. (2018).

The application of different ions as another successful method for promoting mesenchymal cell proliferation, has been supported by articles about in vivo studies: Zhou et al. (2016), Yu et al. (2017).

Modification of nanotubes is supported as a successful technique in activating mesenchymal cells, based on in vitro studies, at published articles: Manfredi et al. (2016), Calzado-Martin et al. (2011), Wei et al. (2019), Singhatanadgit et al. (2019), Kwon and Park (2018).

Attempts have also been made to apply various substances to stimulate mesenchymal cell proliferation.The articles are as follows:

- Sagomonyants et al. (2011)
- Maleki-Ghaleh et al. (2015)
- Yang et al. (2015)
- Hao et al. (2016)
- He et al. (2016)
- Zhou et al. (2017)
- Ping et al. (2017)
- Li et al. (2017)
- Zheng et al. (2017)
- Tsuchiya et al. (2018)
- Shao et al. (2018)
- Chen et al. (2018)
- Deng et al. (2018).

An et al. (2018) published the article about the modification of implant surface, after application of ultrasound, at the differentiation of mesenchymal cells.

For the evaluation of implant success criteria which have been used over time from the beginning of the first presentations, have increased or decreased weight at clinical values of their evaluation (Karthik et al. 2013). The initial criteria are mobility of the implant and marginal radiolucency at dental x-ray. Mobility is the basic criterion for assessing implant failure. As for natural teeth, the mobility of the implant is assessed by the mobility caused by pushing the tail of an instrument and transmitting this push to the fingertip placed above the tooth. This mobility, even if it is at level of 1 mm, it indicates the lack of osteointegration. The greater the mobility expressed in mm, at vestibuilo-oral direction, the more aggravated the clinical situation about osteointegration; which reaches the most visible values when the mobility is also in the vertical direction. Osteointegration is accompanied by proper radiography, which does not show the presence of radiolucency around the implant, the same assessment as for natural teeth. If in natural teeth the main sign of peri-inflammation is the absence of the lamina dura, at the implant surface, the sign of periimplantitis is precisely the presence of radiolucency around the implant. The marginal bone resorption is predetermined by Karthik et al. (2013) For Banemark at osteointegrated implants it was 1.5 mm for the first year, followed by a bone loss of 0.1 mm per year. The average bone loss of 0.2 mm is accepted as a success criterion (Adell et al. 1981).

Coherent concepts for implant success evaluation are: fixed gingival width, type of used suture, current patient's medical status, smoking, implant width. Early concepts are the basic concepts of implant success evaluation then the following concepts were added to them, which seem to play a vital role at long-term clinical success of the placed implant (Karthik et al. 2013).

The width of fixed gingiva, which is known that fluctuate at natural teeth within anatomical limits, depends on the location of tooth at the dental arch. The fixed gingiva is more reduced to the frenulum fixation area and relatively affects the occurrence of gingival recession, as each frenulum may have a muscle fiber embedded within it. This muscle fiber exerts force on the frenulum fixation area, reducing the width of the fixed gingiva, and thus reducing the masticatory function of this gingiva (Heta and Robo 2018). If we talk about implants, they have no clinical success when adhent gingiva is less than or equal to 2 mm. Other studies have shown that the absence or presence of a thin fixed masticatory gingiva, accompanied by bleeding during probing, is another indication of the possibility of greater bone loss (Bouri et al. 2008).

The type of used suture is another element of evaluation. Silk sutures have less potential for bacterial colonization than any other suture, thus minimizing odontogenic infections (Banche et al. 2007). The use of polylactin 910 has a high incidence of early implant loss (Banche et al. 2007).

The medical conditions of the patient's current condition are among the first elements that affect the early loss of the implant. Recent studies support this fact (Klokkevold and Han 2007). Despite the suggestion that type 2 diabetes has side effects on implant clinical survival, there is no conclusive evidence (Koldsland et al. 2009).

Smoking is another element that influences clinical implant success. It is known that smoking causes an increase of gingival fluid that effects the gingival tissues, at local level, but also at systemic level (Robo et al. 2017). There is an evidence that smoking influences osteointegration depending on the daily smoking dose (Alsaadi et al. 2007). Implant width: according to one study, short, thin implants initiate early implant loss (Olate et al. 2010). One possible explanation is that narrow, short implants are placed in areas with minimal bone volume (Baqain et al. 2012).

Genetic and immunological markers are thought to be a diagnostic element in the assessment of implant failure. TNF-alpha and IL-beta released by stimulation of titanium as a material are significantly at higher level than in patients with implant failure (Jacobi-Gresser et al. 2013; Vendramini et al. 2021; Angelopoulos et al. 2022; Pang et al. 2021; Sordi et al. 2021).

Another element that can affect implant success has to do with the radiation of the area where the implant is placed and whether there is a graft placement (Zhang et al. 2021; Marconi et al. 2021). Based on a study, the implant success rate was 89.2% in cases of fibula-graft placement and the success rate was 87.18% at case of irradiated mandible, with the use of hyperbaric oxygen therapy (Brånemark et al. 1999; Branemark et al. 2001).

## Conclusions

Modification of implant surfaces with the aim of further promoting of proliferation of mesenchymal cells adjacent to these surfaces to achieve the “so needed” osteointegration in implant stability has always been a priority area of research in implantology. It is important to note that from the literature review, all modifications and various modification methods are successful in stimulating mesenchymal cells. It cannot be said that one of these methods is superior to the other since scientific research is significantly supported and subsidized by manufacturing companies of various implant type. The clinical success of an implant depends on factors related to the implementation of the implant treatment protocol and factors of the patient's oral and systemic health, the rest of how the implant surfaces are modified, will serve as an added plus to the clinical expectation.

## Data Availability

The datasets analyzed during the current study are available from the corresponding author.
